# Synthesis and characterization of fluorinated polyacrylate latex emulsified with novel surfactants

**DOI:** 10.1080/15685551.2016.1231040

**Published:** 2016-09-30

**Authors:** Cuifeng Zhang, Tingting Xu, Zhongbin Bao, Lijun Chen

**Affiliations:** ^a^ School of Chemical Engineering, Zhejiang University of Technology, Hangzhou, China

**Keywords:** Fluorinated polyacrylate latex, semi-continuous seeded emulsion polymerization, sodium lauryl glutamate

## Abstract

The fluorinated polyacrylate latex were successfully prepared with semi- continuous seeded emulsion polymerization of butyl acrylate (BA), methyl methacrylate (MMA) and hexafluorobutyl methacrylate (HFMA) which was initiated with potassium persulfate (KPS) initiator and emulsified with the novel mixed surfactants of sodium lauryl glutamate (SLG) and alkylphenol ethoxylates (OP-10). The structure of the resultant latex was confirmed by Fourier transform infrared spectroscopy (FTIR). The particle size of the latex was measured by Zetatrac dynamic light scattering detector. The film of latex was tested by differential scanning calorimetry (DSC), thermogravimetric analysis (TGA) and contact angle (CA). The optimum conditions of preparing the novel fluorinated polyacrylate latex are optimized and the results are as follows: the amount of emulsifiers is 4.0%; mass ratio of SLG to OP-10 is 1:1, the amount of the initiator is 0.6%. The mass ratio of MMA to BA is 1:1 and the amount of HFMA is 7.0%. In this case, the conversion is high and the polymerization stability is good. In addition, the water resistance and thermal properties of the latex films were improved significantly in comparison with the film of the latex prepared without the fluorinated monomer.

## 1 Introduction

Emulsion polymerization has been widely used in producing not only environmental friendly copolymers but also organic-inorganic hybrids.[1] In recent years, the acrylate emulsions have attracted a great deal of academic and industrial interests in the field of material science because of its superior performance such as good weather resistance film-forming property, adhesion, transparency, mechanical properties and so on.[2–5] However, their drawbacks such as poor stain resistance, solvent resistance and water resistance limit their further application. Fluorinated polymers present many excellent properties such as good adhesion to matrices, weather resistance and high thermal stability because of the low polarizability and the strong electronegativity of fluorine atom. Thus, some properties of the latex are improved greatly when fluorine-containing entities are incorporated into the structure of polyacrylate.[6–8]

**Scheme 1.**
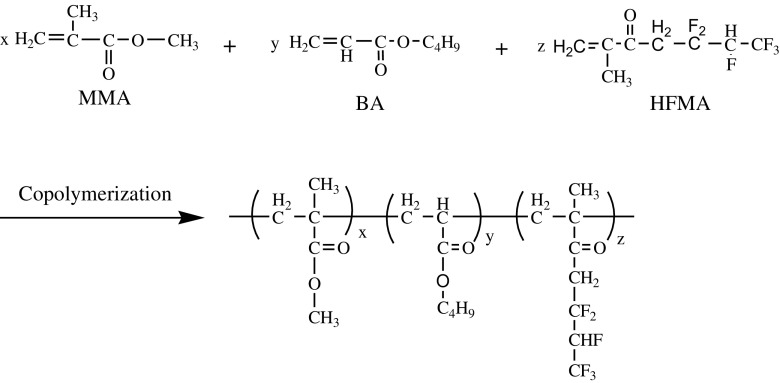
Synthetic pathway of preparing fluorinated polyacrylate latex.

Emulsifiers, which can stabilize the latex, play an important role in emulsion polymerization. Amino acids surfactants were a type of the environmental friendly surfactants based on biological substance. Usually, the mixed surfactants of the anionic surfactant and nonionic surfactant are used to be the emulsifiers in the emulsion polymerization, which can produce synergistic effect and improve emulsion stability and adsorption fastness of the surfactnat on the latex particle.[9] At present, application of amino acids surfactants in preparing the fluorinated polyacrylate latex has not been reported in the open literatures. In this work, the fluorinated polyacrylate latex was prepared when the mixed monomers including the fluorinated monomer were initiated with KPS and emulsified with the mixed surfactants of the SLG and OP-10. The synthetic pathway was given in Scheme 1. The emphasis is put in the present work on optimizing the recipe of preparing the acrylic polymer latex and its characterization. In addition, the influence of the amount of hexafluorobutyl methacrylate (HFMA) on the properties of the copolymer is investigated in detail.

## 2. Experimental

### 2.1. Materials

Methyl methacrylate (MMA) and butyl acrylate (BA), which were analytically pure, were purchased from Shanghai Chemical Reagents Supply Procurement of Five Chemical Plants (China) and were distilled under reduced pressure prior to polymerization. Hexafluorobutyl methacrylate (HFMA), which was industrial grade, was obtained from Harbin Xeogia Fluorine-silicon Material Co Ltd (China). Alkylphenol ethoxylates (OP-10), which were industrial grade, were obtained from Shanghai Minchen Chemical Co Ltd (China). SLG, which was industrial grade, was supplied by Guangzhou Bafeorii Chemical Co Ltd (China). Potassium persulphate (KPS), which was chemically pure, was obtained from Shanghai United Initiators Co Ltd (China). The water used in the experiment was de-ionized.

### 2.2. Preparation of fluorinated polyacrylate latex

The fluorinated polyacrylate latex was synthesized by the semi-continuous seeded emulsion polymerization. In a typical experiment, a 250 ml four-neck flask equipped with a mechanical stirrer, a reflux condenser, and two dropping funnels and heated by the water bath, the rotational rate of the mechanical stirrer was adjusted to 200 r/min. Firstly, all the emulsifiers of SLG and alkylphenol ethoxylates (OP-10) and 40.00 g of de-ionized water were introduced into the flask. Then 10% (wt%) of initiator solution and mixed monomers, which were composed of 13.95 g of MMA, 13.95 g of BA and 2.1 g of HFMA, were dropped into the flask under stirring within 15 min when the temperature was raised to 80 °C. Thus, the seeded latex was obtained after the reaction was maintained for another 15 min. The rest of the mixed monomers and initiator solution were charged into the reactor within 3 h by two separate dropping funnels, respectively. The temperature was raised to 90 °C and maintained for another 45 min after the mixed monomers and initiator solution were dropped completely. Finally, the latex was cooled down to 40 °C. The latex in the flask was filtered to separate coagulate. Thus, the fluorinated polyacrylate latex was obtained. The recipes of preparing the fluorinated polyacryate latex were listed in Table 1.

**Table 1.** Basic recipe of preparing emulsion.

Ingredients	Amount/g	Ingredients	Amount/g
MMA	13.95	OP-10	0.6
BA	13.95	SLG	0.6
HFMA	2.1	De-ionized water	70
KPS	0.18		

### 2.3. Characterizations

The chemical structure of the latex film was characterized by Fourier transform infrared spectroscopy (FTIR, Thermo Nicolet infrared AVATAR370, USA) in a range of 4000–700 cm^−1^. Glass transition temperature (*T*_g_) of copolymers was obtained by the differential scanning calorimetry (DSC Q100, USA). The temperature range was from −60 to 50 °C at a heating rate of 10 °C/min with N_2_ protection. Thermogravimetric analysis (TGA) analyzer (Q50, USA) was used to characterize the thermal stability of copolymers. The heat flow was recorded at heating rate of 10 °C/min from 40 to 500 °C. The average particle size of the latexes was determined by Zetatrac dynamic light scattering detector (Microtrac Limited Corporation, USA) at 25 °C. Contact angle (CA) of water-drop on latex film of copolymer was measured with DataPhysics contact angle meter (OCA-20, Germany). Water absorption of film was determined according to the gravimetric analysis and calculated via the Equation (1):

(1) $$w_{\text{t}}(\%)=\left(m_{1}-m_{0}\right)/m_{0}\times 100\%,$$

where *w*_t_ was the water absorption, *m*_1_ and *m*_0_ was the mass of wet film and dry film, respectively. The monomer conversion and gel percentage were also measured with gravimetric analysis. Conversion percentage was calculated according to the following Equation (2):

(2) $$X\left(\%\right)=\frac{\left(w_{2}-w_{0}\right)/\left(w_{1}-w_{0}\right)-A}{B}\times 100\%,$$

where *X* is the conversion ratio; *w*_2_ is the weight of the dried colloid and bottle; *w*_1_ was the weight of the colloid and bottle; *w*_0_ was the weight of the weighing bottle; *A* is the weight percent of the total non-volatile ingredients in the recipe. *B* is the weight percent of the total monomer in the recipe. Gel percentage of the reaction was calculated according to the following Equation (3):

(3) $$Y_{\text{t}}\left(\%\right)=m_{1}/m_{0}\times 100\%,$$

where *Y*_t_ is the gel percentage, *m*_1_ is the weight of coagulum that was measured by collecting the solid deposited on the stirrer, reactor walls, and by residual of filtered latex, *m*_0_ is the weight of the total monomer in the recipe. The mechanical stability of the emulsion was tested by the centrifugal machine with the rotation speed of 4000 r.min^−1^ with 30 min. Ca^2+^ stability test is carried out by adding 4 ml CaCl_2_ aqueous solution (0.5 wt%) into 16 ml latex in a test tube and it was left standing for 48 h at room temperature to examine the coagulation.

## 3. Results and discussion

### 3.1. FTIR and DSC film analysis of the films

**Figure 1.**
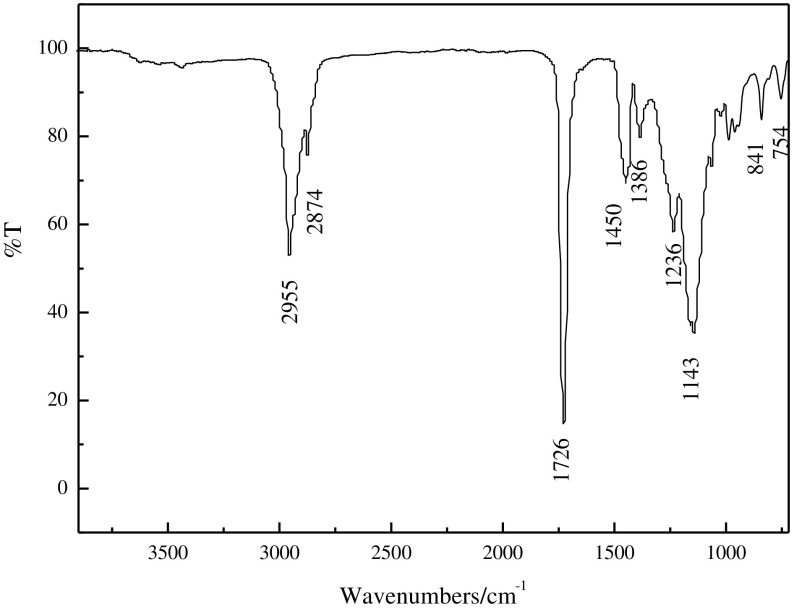
FTIR of film.

Figure 1 shows the FTIR spectrum of the film of the fluorinated polyacrylate latx. The strong absorption band appearing at 2955 and 2874 cm^−1^ are corresponded to the characteristic stretching peaks of C–H (CH_3_, CH_2_) within the butyl acrylate segment. The band at 1726 cm^−1^ is attributed to the stretching vibration of the ester group (C=O) in (meth)acrylate. 1450 cm^−1^ is the bending vibration peak of –CH_2_– and 1385 cm^−1^ was the flexural vibration peak of C–H in –CH_3_. The absorption bands corresponding to the stretching vibration of C–F at 1236 cm^−1^ were also observed. 1143 cm^−1^ was the stretching vibration peak of C–H. A series of absorption bands associated with the C–O bond at 841 cm^−1^ were identified. As well as the absorption peak at 754 cm^−1^ is assigned to the stretching vibration of C–F ultimately. It can be seen that the stretching vibration of C=C disappears within the range of 1500–1700 cm^−1^. The FTIR spectrum reveals that all the monomers have been introduced into the emulsion particles as desired through semi-continuous seeded emulsion polymerization.

**Figure 2.**
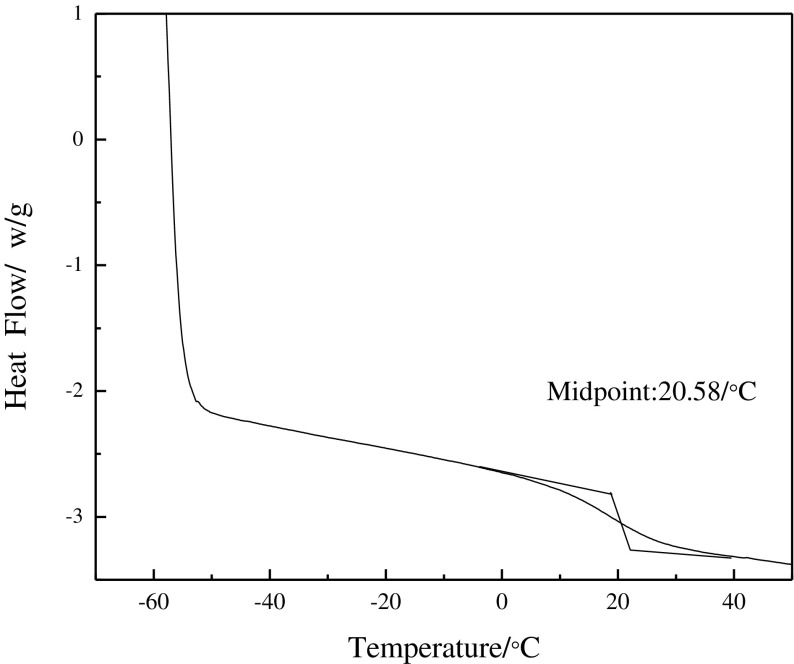
DSC analysis of film.

The DSC curve of the film of the fluorinated polyacrylate latex is presented in Figure 2. It can seen that the *T*_g_ of the film is 20.58 °C and is different from those of homopolymer of BA (−56 °C) and MMA (105 °C), which also confirms that the latex has been prepared successfully. Furthermore, there exists only one *T*_g_ for the film, which indicates that the latex is a kind of random copolymer.

### 3.2. Particle size of latex

Effect of the amount of emulsifiers on average particle size of the latex and its distribution is given in Table 2, which indicates that the average diameter of the latex particles is decreased with increased amount of the emulsifiers. In addition, it also can be seen that both particle size and particle size distribution are uniform comparatively. The reasonable explanation may be that the mechanism of micelle nucleation, i.e. the emulsifier is formed to micelles, and the site of the emulsion polymerization is formed after the part of the micelles obtains the free radicals decomposed from the initiator. It has been proved that the amount of the micelles and the probability of forming the reaction center are nearly constant when the amount of the emulsifiers is fixed. There will be more micelles, which will lead to the decrease of particle size the latex when the amount of emulsifiers is increased. Thus, the average particle of the latexes is decreased when the amount of the emulsifiers is increased.

**Table 2.** Effect of amount of emulsifiers on average particle size of latex.

Amount of emulsifier/%	d/nm	PDI
2.0	305.2	0.091
3.0	197.9	0.035
4.0	101.4	0.064
5.0	85.31	0.120
6.0	77.12	0.095
7.0	72.18	0.112
8.0	66.37	0.130

### 3.3. Influence of mass ratio of monomer on properties of latex

**Figure 3.**
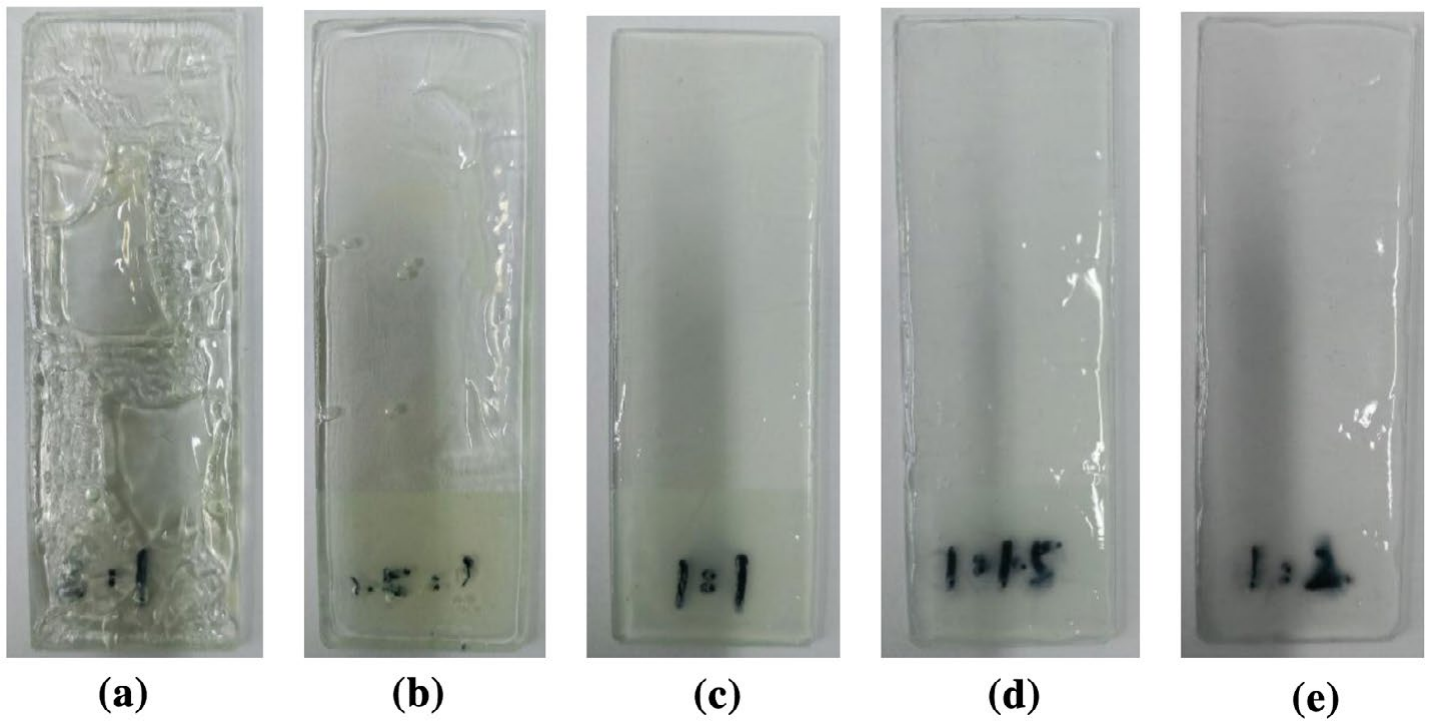
Effect of mass ratio of monomer on appearance of film (a) m(MMA):m(BA)=2:1; (b) m(MMA):m(BA)=1.5:1; (c) m(MMA):m(BA)=1:1; (d) m(MMA):m(BA)=1:1.5; (e) m(MMA):m(BA)=1:2.

Influence of different mass ratios of monomer on properties of latex is shown in Table 3. It can be seen that all conversion rate is more than 98%. And the results indicate that the varied mass ratio of the monomer has a slight effect on the conversion percentage and coagulation percentage. The influence of varied amount of mass ratio of monomers on the properties of the film is given in Figure 3, which indicates that the film is moderately hard when the mass ratio of MMA to BA is 1:1. The above phenomenon can be explained by the further analysis of the theoretical value of the glass transition temperature. According to the fox Equation (4):

(4) $$\frac{1}{T_{g}}=\frac{w_{1}}{T_{1}}+\frac{w_{2}}{T_{2}}+......+\frac{w_{n}}{T_{n}},$$

**Table 3.** Influence of mass ratio of monomer on properties of latex.

Mass ratio of MMA to BA	2:1	1.5:1	1:1	1.5:1	1:2
Conversion percentage/%	99.73	99.98	98.01	99.03	99.26
Coagulation percentage/%	0.39	0.37	0.16	0.19	0.29
Appearance of film	Hard and brittle	Hard	Moderate	Soft	Sticky
Appearance of latex	●	●	●	●	●
Mechanical stability	√	√	√	√	√

Notes: ● stands for appearance of the emulsion is translucent with blue light.

√ stands for emulsion is stability.

where *T*_g_ is the glass transition temperature of copolymers; *w*_n_ is mass percent of monomer in copolymer composition, *T*_n_ is the glass transition temperature of the homopolymer of the monomer. Usually, soft monomers bring the film of the latex with a certain degree of flexibility and improve the water resistance and stability of the latex moderately. However, too much soft monomer makes the film-forming properties decrease, which cause the film surface to be sticky. In addition, the film-forming properties are improved with the increase of amount of hard monomers. However, too much hard monomers cause the water resistance of the film to be decreased and the hardness of the film to be increased. In view of the flexibility and hardness of the film, the mass ratio of MMA to BA is 1:1 in this study.

### 3.4. Influence of amount of emulsifier and mass ratio on properties of latex

Emulsifier plays an important role in emulsion polymerization, which has an obvious effect on the properties of the emulsion such as stability of emulsion, the formation of the particles, polymerization rate and latex particle size distribution and so on. It is difficult to form a stable emulsion if there is no emulsifier in emulsion polymerization. Usually, it is difficult to remove the emulsifier in the resultant emulsion. Thus, the emulsifier still exists in the formed film of the emulsion. The influence of the varied amount of the emulsifier on the properties of the latex is shown in Table 4. It can be seen that the appearance of latex is milky white when the amount of the emulsifier is fewer. However, the appearance of latex is translucent with blue light when the amount of the emulsifier is increased further. The reason for changes of the appearance of latex is that the particle size of the latex is bigger when the amount of emulsifier is fewer. However, the average size of the latex particles is decreased with the increased amount of emulsifiers. In addition, Table 4 also indicates that the varied amount of the emulsifier has no obvious effect on the coagulation percentage. But conversion percentage is increased gradually with the increased concentration of the emulsifier when it is less than 5% (wt%). Furthermore, it can be found that the coagulation percentage is the minimum when the concentration of emulsifier is 5%. The reasonable explanation may be that emulsifiers can scatter monomers into small droplets and form more micelles that provide the site of polymerization. When the emulsifier content is fewer, the amount of emulsifier cannot reach the critical micelle concentration, which causes the monomers not to be emulsified completely. Thus, the part of the monomer cannot enter micelles to participate in the reaction thus causing low conversion rate. In addition, the stability of emulsion polymerization and the micelle concentration are increased with increased amount of emulsifier, which results in obtaining more latex particles and more reactive centers thus causing conversion to be increased. Nevertheless, the latex particles will be completely covered when the amount of emulsifier is excessive. It is difficult for the free radicals to enter inside of latex particles, which leads to lower conversion.[10] Usually, the mixture of nonionic and anionic surfactant is often adopted in practice. The use of the mixture of nonionic and anionic surfactant can improve the cloud point of nonionic surfactant greatly and make the surfactant molecules be alternately absorbed onto the surface of latex particles, which reduce static repulsion among the ions on the same latex particle and charge density on the surface of latex particles. In addition, anionic surfactants are absorbed onto the surface of latex particles which form surface electronegative layer. Zeta potential is formed from close layer to system body and their static repulsion keeps the system stable. Furthermore, during the emulsion polymerization, nonionic surfactants are absorbed onto the surface of the latex particles which form the elastic interface film. It is with the film that the coagulation of the latex particles is prevented. In view of the stability of the emulsion and conversion, the amount of the emulsifier is 4.0% in this work, the suitable mass ratio of SLG to OP-10 is 1:1.

**Table 4.** Influence of amount of emulsifier and its mass ratio on properties of emulsion.

Amount of emulsifier/%	Mass ratio of SLG to OP-10	Conversion percentage/%	Coagulation percentage/%	Appearance of latex	Mechanical stability	Ca^2+^ stability
2.0	1.0:1.0	94.02	1.39	▲	√	√
3.0	1.5:1.5	95.47	1.17	▲	√	√
4.0	2.0:2.0	96.56	0.91	●	√	√
5.0	2.5:2.5	98.00	0.67	●	√	■
6.0	3.0:3.0	97.36	0.71	●	√	■
7.0	3.5:3.5	97.01	0.60	●	√	■
8.0	4.0:4.0	96.75	0.87	●	√	■

Notes: ▲ means that the appearance of the latex is milky white.

● means that the appearance of the latex is translucent with blue light.

√ stands for the good stability of latex.

■ stands for the poor stability of latex.

### 3.5. Influence of amount of initiator on conversion percentage and coagulation percentage

**Figure 4.**
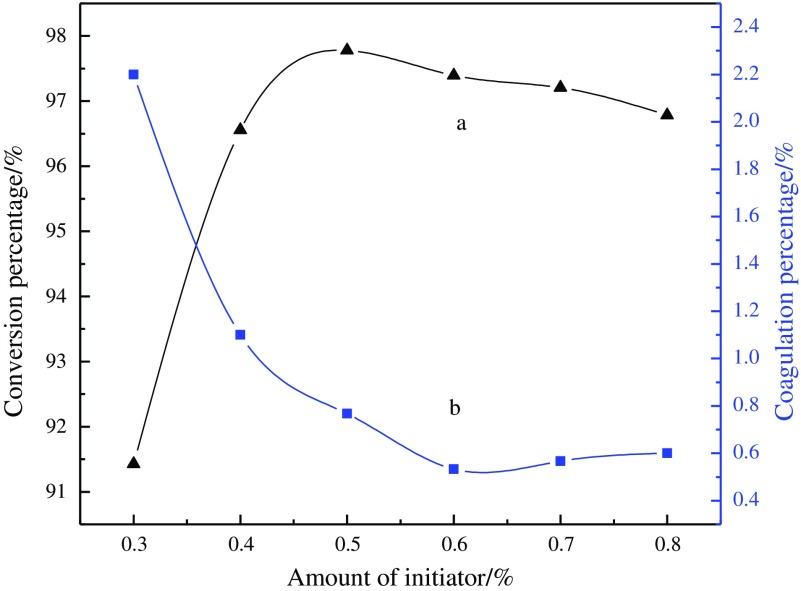
Influence of amount of initiator on conversion percentage (a) coagulum; (b) percentage.

The effect of the varied amount of the initiator on the conversion percentage and coagulation percentage is given in Figure 4. Figure 4 shows that the conversion is increased with the increased concentration of initiator and the conversion is the maximum when the amount of the initiator is 0.5%. On the contrary, the coagulation percentage is decreased with the increase of the amount of the initiator. This phenomenon may be attributed to the fact that the increased amount of initiator will lead to more free radical that causes the increase of conversion and decrease of coagulation. However, the polymerization is so fast that the heat of reaction is not easy to be expelled when the amount of initiator is excessive, which makes the polymerization system be difficult to control and the stability of the latex be also reduced. Thus, the optimum amount of the initiator is 0.6% in this study.

### 3.6. TGA of films

**Figure 5.**
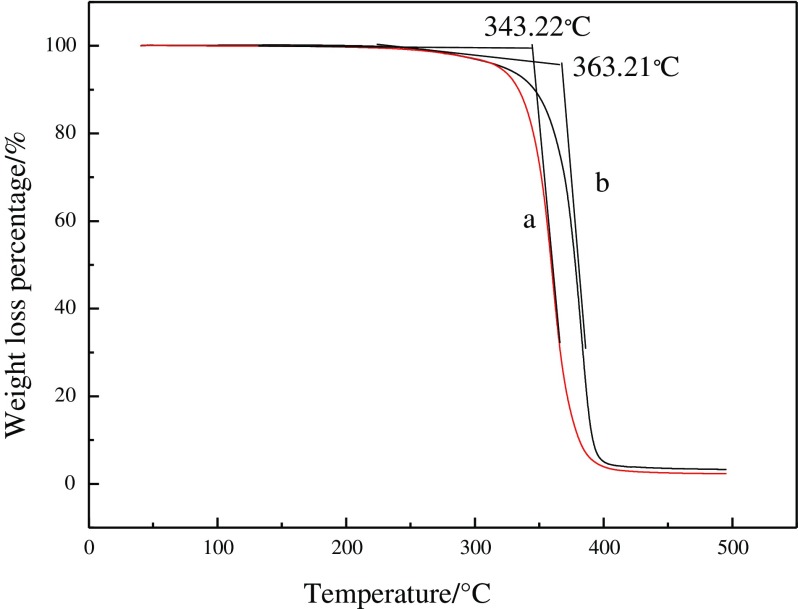
TGA of film (a) acrylate copolymer; (b) acrylate copolymer modified by fluorine monomer.

Thermogravimetry curves of the latex copolymers are shown in Figure 5. It is seen that the decomposing temperature of fluorinated polyacrylate latex is 363.21 °C, which is 20 °C higher than that of the fluorine-free polyacrylate latex. This means that the thermal stability of the fluorinated polyacrylate latex has been improved significantly. The obvious increase of the thermal stability of the film is caused by the fact that the fluorine atom has been incorporated into the polymer during the emulsion polymerisation. The bond energy of C–F is higher than that of C–C.

### 3.7. Water absorption and contact angle

**Figure 6.**
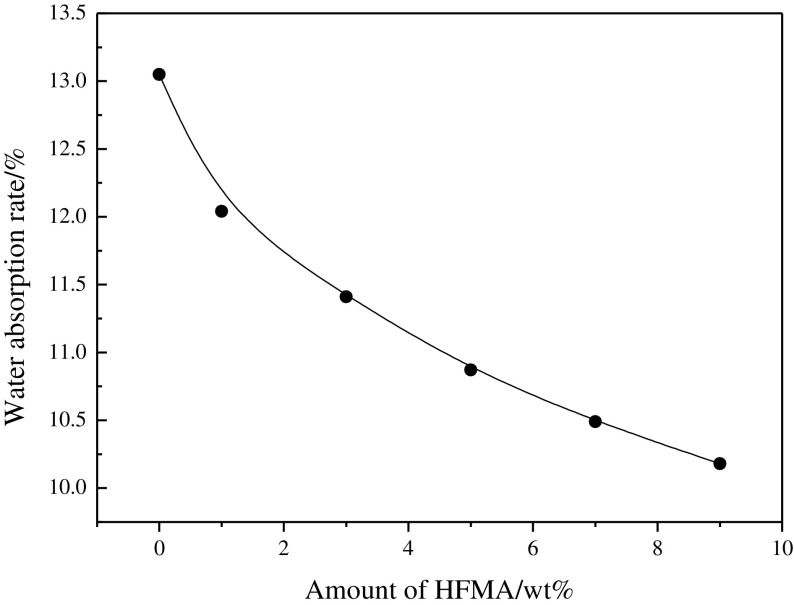
Influence of amount of HFMA on water absorption of film.

**Figure 7.**
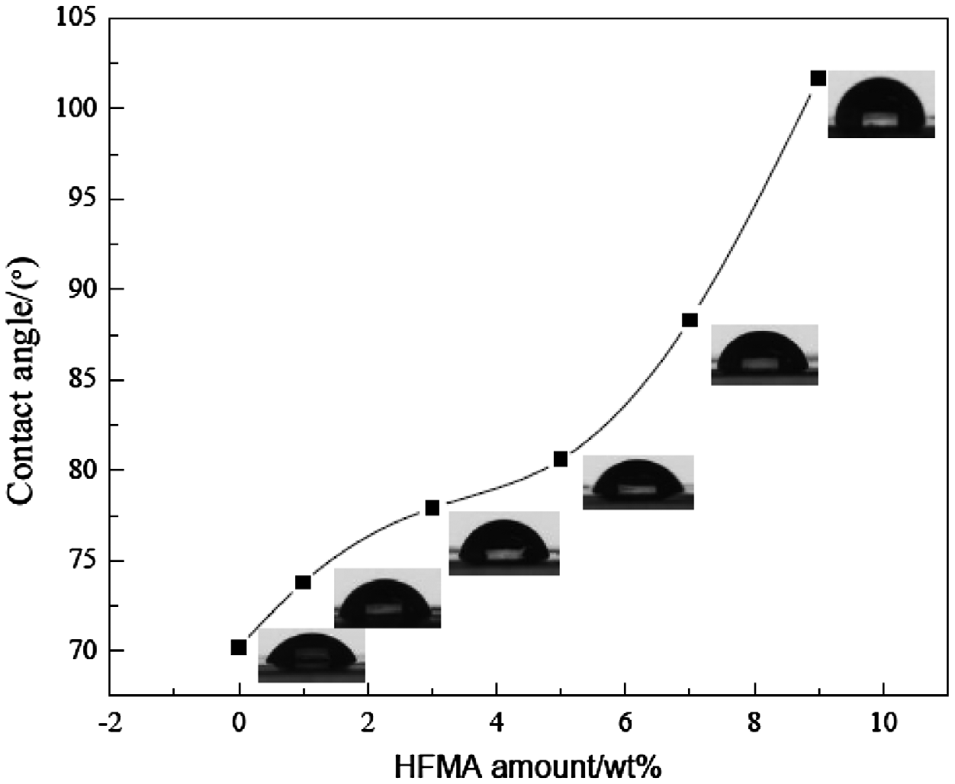
Influence of amount of HFMA on CA.

The influence of the amount of fluorinated monomers on the water absorption and contact angle is presented in Figures 6 and 7, respectively. Figure 6 shows that the water absorption of the latex film is decreased obviously when the fluorinated monomer is introduced into the latex. The reason may be that the smaller surface energy of C–F results in the improvement of the water resistance of the latex film. Usually, the hydrophobicity of the material is influenced significantly by its surface chemical composition and is usually estimated with the water contact angle. Thus, the water contact angle has commonly been used as a criterion for the evaluation of hydrophobic surfaces.[11] The higher contact angle, the better the hydrophobic property is. In Figure 7, it can be seen that the hydrophobic property of fluorine containing emulsion is good and CA is increased with the increased amount of HFMA. These phenomena can be explained by the fact that the fluorinated groups might preferentially migrate to the surface and locate at the interface to minimize the interfacial energy, especially at high temperature circumstances.[12,13] CA is increased clearly owing to the C–F bond is highly hydrophobic, which improves the water resistance of the film.

## 4. Conclusions

The fluorinated latex is successfully prepared with semi-continuous seeded emulsion polymerization of BA, MMA and HFMA in the presence of the mixed emulsifier of SLG and OP-10, which were initiated with KPS. The optimum recipe of preparing the latex is as follows: the amount of emulsifiers is 4.0%; mass ratio of SLG to OP-10 is 1:1; the amount of the initiator is 0.6%. The mass ratio of MMA to BA is 1:1; the amount of HFMA is 7.0%. Heat resistance and hydrophobicity of latex films are improved clearly because of the introduction of fluorine into the polymer.

## Disclosure statement

No potential conflict of interest was reported by the authors.

## Funding

This work has been supported by the Open Project of Zhejiang Provincial Environmental Protection Printing Ink Engineering Technology Research Center [grant number XDF2014GCKF001].
